# Self-Justification Processes Related to Bullying Among Brazilian Adolescents: A Mixed Methods Study

**DOI:** 10.3389/fpsyg.2019.01086

**Published:** 2019-05-15

**Authors:** Wanderlei Abadio de Oliveira, Simona C. S. Caravita, Barbara Colombo, Elisa Donghi, Jorge Luiz da Silva, Marta Angélica Iossi Silva

**Affiliations:** ^1^Faculty of Philosophy, Sciences and Letters of Ribeirão Preto, University of São Paulo, Ribeirão Preto, Brazil; ^2^C.R.I.d.e.e., Department of Psychology, Catholic University of the Sacred Heart, Milan, Italy; ^3^Psychology, Champlain College, Burlington, VT, United States; ^4^Department of Health Promotion, University of Franca, Franca, Brazil; ^5^Ribeirão Preto College of Nursing, University of São Paulo, Ribeirão Preto, Brazil

**Keywords:** bullying, moral disengagement, Brazil, adolescence, mixed method design

## Abstract

This study aims to investigate the associations between bullying and moral disengagement in a Brazilian sample, using a mixed method design. Two-thousand three hundred and thirty-four adolescents (11–19 years; 42.9% girls) answered self-report measures on bullying and moral disengagement in response to bullying situations. Fifty-five participants were randomly selected and interviewed on their experiences on bullying at school. Results allowed to identify specific mechanisms of moral disengagement associated with bullying behavior among Brazilian adolescents. Qualitative analysis highlighted how moral disengagement mechanisms were spontaneously used by the adolescents to explain both the bullying and the bystander behaviors. Findings support the relevance of moral disengagement mechanisms in explaining bullying behaviors. The value of addressing these mechanisms when designing anti-bullying interventions is discussed.

## Introduction

Bullying is a growing phenomenon across over world and it is generally believed to constitute a serious risk for the whole society. Internationally, bullying has been widely studied. The scientific literature reports a consistent picture of bullying across countries, and in Western countries, along with other maladaptive behaviors, bullying is becoming a prominent problem among adolescents. Nevertheless, this phenomenon and the psychological processes that increase the risk for bullying, are still under studied in Brazil. In particular, the literature identifies in self-justification processes of moral disengagement a critical element enhancing bullying (Caravita et al., 2012). Moral disengagement is typically assessed by means of quantitative measures. Nevertheless, even if solid, the quantitative approach may be not enough to properly investigate moral disengagement and its associations with bullying. The quantitative approach allows to highlight a correlation between the tendency to use moral disengagement and the actual bullying behavior, but fails to explore the reasons behind the correlations that can be derived by studying the spontaneous use of self-justifications in narratives of bullying. The main objective of our study was to explore the relations between moral disengagement processes and bullying/passive bystander behavior by means of quantitative and qualitative data and involving a sample of Brazilian adolescents (aged 10–19 years).

Bullying can be defined as a subtype of aggressive behavior, which is goal-directed, deliberate and related to peer-group dynamics (e.g., Caravita et al., 2011b). This phenomenon is identified more as a proactive than a reactive form of aggression (Camodeca and Goossens, 2005), characterized by intentional reiteration over time of the attacks and by an imbalance of power between the victim or the victims and the bully or the bullies (Olweus, 2013). Even if some recent literature on gender differences in association with bullying and victimization reported mixed results (e.g., Veenstra et al., 2005; Carbone-Lopez et al., 2010; Crapanzano et al., 2011), the profile of the bully has been typically associated more with the male than the female gender (Psalti, 2012). Boys have been suggested to perform actions of direct bullying (i.e., physical attacks, punching, and kicking), more frequently than girls. On the other hand, girls tend to perform indirect bullying, (i.e., forms of social isolation, intentional exclusion from the peer group, and slander), more frequently than boys (Veenstra et al., 2005; Psalti, 2012).

Literature also reports that, by performing a bullying behavior, the bully aims to affirm their supremacy on the victims, to provoke physical or psychological harm to them, despite the absence of any provocation (Silva et al., 2008). These specific characteristics of bullying behavior define it as an immoral act, because it violates the rights of freedom, security, and education of children and adolescents during the school years, and it is intentionally aimed at harming the victim (Hymel et al., 2010).

Bullying is a social phenomenon, which takes place in a relatively stable peer group, usually the classmates. Furthermore, the bully often gets support from other group members, who assume the roles of assistants or supporters of the bully (Salmivalli et al., 1996).

Moral disengagement processes (i.e., psychological mechanisms of self-justification of behaviors that are acted in contrast of personal values; Bandura, 2002) have been often associated with an increased risk for bullying in international studies (Caravita et al., 2012, 2014; Sijtsema et al., 2014; Thornberg and Jungert, 2014; for a metanalysis on bullying and aggressive behavior, including bullying, see Gini et al., 2014), where researchers have underlined the usefulness of the moral disengagement theory to explain this negative behavior among children and adolescents (Hymel et al., 2010). Among the several studies on the association between moral disengagement and bullying (e.g., Caravita et al., 2012; Robson and Witenberg, 2013; Sijtsema et al., 2014), Hymel et al. (2005) confirmed the existence of a positive association between bullying and moral disengagement in a study involving 494 Canadian adolescents. Accordingly, Caravita et al. (2014) showed that moral disengagement is associated prospectively with bullying, and that self-justification processes are socialized among friends in early adolescence.

When considering possible gender-differences in relation to moral disengagement, literature provides evidence that boys tend to morally disengage more than girls (Bandura et al., 2001; Almeida et al., 2009; Obermann, 2011; Perren et al., 2012; Thornberg and Jungert, 2013), and that moral disengagement tends to decline with age (Shulman et al., 2011). In a study examining the eight mechanisms of moral disengagement in a sample of 372 Swedish adolescents (10–14-year-old), boys showed to use significantly higher levels of moral justification, euphemistic labeling, diffusion of responsibility, consequence distortions, and victim blame, than girls did (Thornberg and Jungert, 2014). In the same study, younger students and girls were more likely to defend the bullied peers than older students and boys; furthermore, diffusion of responsibility and blaming the victim mechanisms were significantly and negatively related to defending the bullied peers (Thornberg and Jungert, 2014).

The literature highlights how bystanders of bullying can also apply moral disengagement mechanisms to justify their behavior when witnessing bullying. Indeed, some students feel that defending the victim is morally correct, but they also report not defending the bullied victim because they fear the reaction of the bullies or the peer group. Hence, they can use moral disengagement processes to self-justify their withdrawing from bullying situations. For instance, in a study involving 660 Danish adolescents (11–14 years), who have been identified as unconcerned bystanders since they reported witnessed peers being bullied without feeling responsible, results showed how they tended to use moral disengagement (Obermann, 2011). To be more precise, unconcerned bystanders reported higher levels of moral disengagement when compared to peers who were likely to help the victims in bullying episodes (defending bystanders), and to bystanders who did nothing to help the bullied peers but who felt guilty about it (guilty bystanders) (Obermann, 2011). We can hence derive that moral disengagement mechanisms play an important role in bullying situations and that they need to be addressed in anti-bullying interventions.

From a methodological standpoint, most of the studies investigating the relation between moral disengagement and bullying assessed moral disengagement by means of quantitative measures assessing the general tendency to use self-justification processes when evaluating transgressive conducts (e.g., Obermann, 2011; Caravita et al., 2012). This approach allows assessing individual’s disposition to use of moral disengagement but does not allow to catch the actual use of self-justification processes when the person fronts their own transgressive behavior. Therefore, using different approaches, such as qualitative or mixed methods, may allow to explore with more accuracy the process of self-justification in bullying situations (Thornberg et al., 2012a). Similar approaches may also allow to catch the spontaneous use of moral disengagement processes to self-justify the actual bullying behavior in order to avoid feeling guilty. Following this line of reasoning, no study, to our knowledge, has investigated moral disengagement mechanisms and their spontaneous use in association to the bullying behavior among Brazilian student population.

Because of the studies on bullying in Brazil being relatively recent, data on this specific topic are still scarce. The first studies on bullying in Brazil date back to early 2000, and they mainly focused on possible interventions and on the role of teachers in preventing and contrasting bullying behaviors, without investigating specific elements and correlates of bullying in the Brazilian context (Lopes Neto, 2005; Silva et al., 2008, 2014). Yet, bullying appears to be quite present in the country: a study run on a national sample of 104,109 Brazilian students, 28% of the participants have been found to be involved in bullying as bullies and victims (Oliveira et al., 2015). More recently, a few studies have started to investigate the possible correlates of involvement in bullying among Brazilian students, focusing mainly on family dimensions and individual characteristics of bullies and victims. In particular, a recent study involving a sample of 2,600 adolescents (age *M* = 15 years) explored adolescents’ behavioral self-regulation, assessed through responses to hypothetical bullying-related situations. In this study, bystanders have been found to be more morally engaged (i.e., more empathic and motivated to help the victims) than bullies (Tognetta and Rosário, 2013). This line of research has shed some light on the complexity of bullying among Brazilian students too, but, overall, studies exploring possible risk factors and correlates of bullying behavior in the reality of Brazil are still very limited.

Following the discussion of recent literature presented above, it emerges clearly how, till now, no study has investigated moral disengagement processes as possible correlates of bullying behavior among Brazilian students. Moreover, to our knowledge, in the international literature mixed-method designs (combining quantitative and qualitative data) have not been used to examine the relations between moral disengagement, and the involvement in bullying with the role of either bully or passive bystander. Nevertheless, mixed-method studies would allow to investigate more deeply these associations by overcoming some of the limitations of the self-reported studies discussed in the previous paragraphs.

### The Present Study

Main objective of this study was exploring the relations between moral disengagement processes, bullying and passive bystander behavior by means of quantitative and qualitative data, involving a sample of Brazilian adolescents (aged 10–19 years). As a first step, we investigated the association between bullying and moral disengagement processes as derived from quantitative data. We hypothesized that among Brazilian adolescents the use of moral disengagement to self-justify bullying is associated with higher levels of bullying, confirming data reported in international literature.

As a second step, we tested the hypothesis that spontaneous use of moral disengagement mechanisms to explain bullying and the passive bystander behavior could be identified using qualitative data to analyze adolescents’ spontaneous narrative of bullying behaviors. Indeed, when considering the specificity of moral disengagement as intra-psychological processes of self-justification, qualitative data related to personal experiences of involvement in bullying should allow to identify the actual use of these mechanisms better than quantitative data, which are collected by means of self-report measures of moral disengagement as a stable disposition. To test this hypothesis, we examined the narratives of 55 adolescents, who were interviewed on episodes of bullying in which they were involved.

Our third goal was to examine whether distinct associations of the eight moral disengagement mechanisms with involvement in bullying as a bully (both quantitative and qualitative data) and as a passive bystander (qualitative data) exist. Specifically, we hypothesized that moral disengagement processes that are more strictly connected with the group-nature of bullying, such as the diffusion of responsibility mechanism, are more likely to be associated with behaving bullying and with the passive bystander behavior than the other mechanisms are. We also hypothesized that gender could moderate the relations between moral disengagement mechanisms and bullying (both quantitative and qualitative data) and passive bystander (qualitative data) behaviors (Thornberg and Jungert, 2014). Starting from the evidence reported in the literature (e.g., Almeida et al., 2009), we hypothesized that boys would be more prone to use moral disengagement than girls when explaining bullying.

## Materials and Methods

### Participants

Two-thousand three hundred and 34 students (50.6% girls; *M* = 14.50 years, *SD* = 2.01 years) attending to 11 state schools of Uberaba City were involved in the study. Fifty-five students (46.5% girls; age *M* = 15 years, *SD* = 2 years) were extracted, by randomly selecting five students from each school, and participated in the qualitative part of the study. This selection criteria is consistent with the sampling per maximum variation method (Wuest, 2010). The majority of participants lived with both the father and the mother (57.2%). The mother was present in 90% of the students’ families. Teens who reported living with other adults mentioned grandparents, uncles, brothers, cousins, nephews, husbands, or friends.

Uberaba City is one of the biggest urban centers in Southeast Brazil, and the downtown population of the City is of approximately 319,000 inhabitants. According to data and estimates of the Brazilian Institute of Geography and Statistics (2010), 60.8% of the city’s inhabitants declare themselves white and 28.2% declare themselves to be brown. In terms of religious practice, the majority of the inhabitants are Catholics (60.8%) and Spiritists (15.6%) (Brazilian Institute of Geography and Statistics, 2010). The medium income of the population, in the majority of the cases (64.3%), is of up to two minimum salaries (U$504) (Brazilian Institute of Geography and Statistics, 2010). The study participants on the whole, represent this local reality.

The 11 schools included in this study were selected among the 34 public and state schools (strata) of Uberaba City by using the method of probability proportional to size (Szwarcwald and Damacena, 2008). The main feature of this method is that the probability of each school (strata) to be selected for the sample is proportional to the size (i.e., the number of students) of each strata, and depends of the geographical dimensions and organization of the cities (Bolfarine and Bussab, 2005; Szwarcwald and Damacena, 2008). The region where Uberaba City is located has one of the highest rate of population density in Brazil, with high levels of ethnic and cultural diversity, but with a sociodemographic composition that can be compared to the overall country (Ministry of Health, 2013).

The education system in Brazil is organized into three main cycles: kindergarten (0–5 years), elementary school (6–14 years), and high school (from 15 year to adulthood, with no age limit). The elementary school system comprises 9 years (1–9) and high school is organized into a 3 years cycle (1–3). Participants in this study attended grades 6 to 9 (elementary schools) and 1 to 3 (high school) of the selected schools.

### Procedure

The two questionnaires (see Measure section for details) were group-administered in the classrooms between August and September of 2014, during regular school hours. One of the researchers supervised the measure administration, read out the written instructions for each instrument and answered participants’ questions. Another researcher, expert in qualitative methods, conducted individual qualitative semi-structured interviews. The interviews took place in a quiet room in the school building, far away from other students and teachers.

The current study was part of a broader research project, which was approved by the Ethical Committee of the Ribeirão Preto College of Nursing at University of São Paulo (Brazil). A letter describing the objectives and the procedure of the research project was sent to all the students in the 11 schools involved in the study. Participants and their parents were requested to sign and give back the written consent forms accompanying the letter, if participation in the research project was granted by the parents or guardians. Participants and their parents were also informed that they were free to withdraw from the study at any time during and data collection, without providing any justification for this choice. Seventy-one students (3.0% of the contacted pupils) declined to be involved in the study or did not return the informed written consent signed by their parents, and were thus excluded.

### Measures

#### Aggression and Victimization Scale (Cunha et al., 2009)

In order to identify bullies and victims, the self-report measure *Aggression and Victimization Scale* (Cunha et al., 2009) was used. The scale was developed to assess bullying behavior and victimization within the Brazilian school context, from the elementary school through the high school. The measure consists of 18 items, with 10 items assessing bullying behavior (Bullying subscale; α = 0.83), and 8 items assessing being bullied by peers (Victimization subscale; α = 0.85). Respondents are asked, for each item, to evaluate the frequency they behaved or suffered the described situation on a five-points Likert scale (from 1 = never to 5 = almost always). An example from the bullying subscale items is “I teased my classmates”; while an example from the victimization subscale is: “my classmates provoked me.” Since the original version of the scale did not include a definition of bullying, the scale was adapted for the current study, by adding a definition explaining what bullying is in comparison to other forms of aggression (Solberg and Olweus, 2003). The definition was read aloud to participants before administering the scale, and the respondents were asked to refer to this definition while answering the items. This adaptation of the scale was done in accordance to recommendations from the literature on bullying, and to the procedure used for comparable measures for the assessment of bullying behaviors, e.g., the *Olweus’ Bully/Victim Questionnaire* (Solberg and Olweus, 2003). The authors of the *Aggression and Victimization Scale* authorized this modification.

The scale structure was tested in our sample by performing a Confirmatory Factor Analysis (CFA; MPlus 7.0, Muthén and Muthén, 1998–2015) where the 10 items assessing bullying and the 8 items assessing victimization were specified as loading two separate factors. The model obtained adequate fit indexes in the overall group [χ^2^(133) = 848.71, *p* < 0.001, *CFI* = 0.92, *RMSEA* = 0.048] and in each gender group separately [boys: χ^2^(133) = 545.39, *p* < 0.001, *CFI* = 0.91, *RMSEA* = 0.052; girls: χ^2^(133) = 433.75, *p* < 0.001, *CFI* = 0.93, *RMSEA* = 0.044]. A multiple group analysis, where the item intercepts and loadings were fixed to be equal across the gender groups, confirmed the measure scalar invariance for gender [χ^2^(298) = 1197.32, *p* < 0.001, *CFI* = 0.90, *RMSEA* = 0.051]. Only the subscale for bullying was considered in the current study.

#### Moral Disengagement (Caravita et al., 2011a)

The tendency to morally disengage was assessed by administering a self-report questionnaire devised to assess moral disengagement for bullying behaviors. The measure consists of 30 items, and it was developed by adapting the scale by Caprara et al. (1995). The items present moral exoneration statements of bullying conduct. In order to be sure that respondents are referring to bullying while answering, a definition of bullying is provided at the beginning of the questionnaire. Respondents are asked to rate each item on a five-point scale, ranging from 1 (strongly disagree) to 5 (strongly agree); higher scores indicate a higher tendency to engage in that self-justification for bullying. The thirty items are organized in eight subscales assessing the eight moral disengagement mechanisms. Euphemistic labeling, moral justification, consequence distortions, and victim dehumanization are each assessed by four items (e.g., euphemistic labeling: “Hitting annoying classmates is just like giving them a lesson”; moral justification: “Hitting a classmate to defend one’s own friends is right,” consequences distortions: “Children do not feel offended when somebody makes fun of them because this is a way of showing interest in them”; victim dehumanization: “Mistreating a classmate is ok if they behave like a disgusting animal”). Three items assess each of the following mechanisms: advantageous comparison (e.g., “Insulting a classmate is not serious because hitting them is worse”), blaming the victim (e.g., “Children who are mistreated by schoolmates usually do deserve it”), and diffusion of responsibility (e.g., “If all the classmates make fun of a kid, blaming only one of them is not right”). Displacement of responsibility is assessed by five items (e.g., “If kids live in a difficult neighborhood they cannot be blamed if they take it out on their classmates”).

A third-order CFA was performed to test the scale structure. In the model the 30 items were specified as loading eight factors, each factor assessing one of the eight moral disengagement mechanism. The eight factors loaded the four moral disengagement clusters, which loaded the unique factor assessing overall moral disengagement. In the confirmatory factor analysis, three items (“Making fun of a classmate does not really hurt them,” “If kids live in a difficult neighborhood they cannot be blamed if they take it out on their classmates,” “Saying that a classmate is the “teacher’s pet” is not bad because it does not causes damage to them”) did not load the factors according to the theoretical structure of the scale and were then removed from the model. The final model, consisting of 27 items, fitted the data adequately in the overall group [χ^2^(249) = 1233.94, *p* < 0.001, *CFI* = 0.90, *RMSEA* = 0.041], and among boys [χ^2^(249) = 703.93, *p* < 0.001, *CFI* = 0.91, *RMSEA* = 0.043]. The model also obtained adequate fit indices among girls after correlating four pairs of item errors: χ^2^(245) = 697.61, *p* < 0.001, *CFI* = 0.90, *RMSEA* = 0.039. The scale invariance across gender groups was tested by means of multiple group analyses, in which the item intercepts and loadings were fixed to be equal across the gender groups. The multiple group model fit was close to be, but still not adequate [χ^2^(530) = 1607.58, *p* < 0.001, *CFI* = 0.89, *RMSEA* = 0.042], and became fully adequate [χ^2^(524) = 1475.85, *p* < 0.001, *CFI* = 0.90, *RMSEA* = 0.039] after allowing the correlation of three pairs of item errors in the boys group (two of the item error pairs corresponded to two item error pairs already correlated among girls), and freeing three intercepts among boys. Thus, the analysis provided evidence for partial scalar invariance of the measure across the gender groups. For the current study the factors’ scores of the subscales of the eight mechanisms have been estimated starting from this final model and used in the models as measures of the moral disengagement processes. Reliability indexes (Chronbach’s α) for the eight subscales assessing the moral disengagement mechanisms ranged from 0.79 to 0.87.

#### Semi Structured Interview

Narratives about adolescents’ experiences in bullying were recorded using individual semi-structured interviews. This technique was chosen because it allows the respondent to express their opinion, to develop a more personal narrative concerning the interview topic, and to share information about context or social phenomenon better than when responding to quantitative scales (Taylor and de Vocht, 2011). This technique also gives the researcher a higher control over the data collection than other forms of qualitative techniques, such as focus groups (Taylor and de Vocht, 2011). An interview guide was used. Participants were asked to talk about (1) their victimization experiences of school bullying (2) their bullying experiences (3) their perceptions as bystanders. As an example, these were some of the questions used: “Have you ever threatened, mistreated, humiliated, or assaulted another student at school?”; “What did you do?”; “Have you been threatened, humiliated or been the victim of aggression at school?” Some follow-up questions were used to clarify students’ responses (e.g., “How did that happened?,” “Could you tell me more about that?,” “Tell me about it,” “What do you mean?,” “Tell me more,” and “What do you think about that?”). The interviews lasted 12 min on average (time range: from 6 to 26 min). Each interview was audio-recorded and transcribed verbatim.

### Analysis

#### Quantitative Analysis

Structural equation modeling (Maximum Likelihood estimator; Mplus 7.0, Muthén and Muthén, 1998–2015) was utilized to test our hypotheses about the associations of moral disengagement and bullying. A model was specified where the latent score for bullying was regressed on the factor scores (manifest variables) of the eight moral disengagement subscales. Age was included in the model in order to control for its effects. Goodness of fit of the tested models was verified by examining the Chi square index, which needs to be non-significant (*p* > 0.05) for models fitting the data adequately. Since the Chi square index is very sensitive to the size of the sample, becoming significant with large samples, the Comparative Fit Index (CFI) was also examined. The CFI index compares the existing model fit with a null model assuming the independence of the variables in the model, thus evaluating the adaptation of the estimated model to the observed data. The value of CFI is acceptable when equal or superior to 0.90 and good when equal or superior to 0.95. The Root-Mean-Square Error of Approximation (RMSEA) was also computed to estimate the residuals of the data that the model does not explain. For models obtaining adequate fit the RMSEA is equal or less than 0.08; for models with a good fit the RMSEA is equal or inferior to 0.05.

The model was first tested in the overall sample. Then, possible moderation by gender was explored by testing the model in each gender-group separately, and by performing a multiple group analysis where the intercepts and the loadings of the latent factor were constrained to be equal across the two gender groups.

#### Qualitative Analysis

A content analysis was conducted using ATLAS.ti qualitative data analysis software, version 7 (ATLAS.ti, 2015), to identify emerging themes related to mechanisms of moral disengagement. Two researchers, who were Brazilian Portuguese native speakers, as well as experts in the psychological processes related to bullying and morality and in the qualitative analysis procedure, codified the interviews using the software. First, an exhaustive reading of the transcripts of the interviews was conducted to identify the main themes that emerged from the data. Next, the textual information from transcriptions was analyzed, and codes were created until saturation was reached. Then, the coded transcripts were analyzed to explore the issues related to the mechanisms of moral disengagement that spontaneously emerged in participants’ narratives. Quotes illustrating meaning or key messages from the analysis were selected, based on the code count or on how much they were exemplifying the discussion topic. The quotes have been translated into English.

## Results

Means and standard deviations of the manifest and latent variables, along with variable correlations, are displayed in Table 1 for the overall sample, and in Table 2 for boys and girls separately.

**Table 1:** Means, standard deviation, and correlations (overall sample).

	Mean	*SD*	1	2	3	4	5	6	7	8	9	10	11
1. Moral justification	2.37	1.03	–										
2. Euphemistic labeling	2.02	0.94	0.61**	–									
3. Blaming the victim	2.19	0.95	0.43**	0.46**	–								
4. Displacement resp.	2.32	0.90	0.45**	0.47**	0.76**	–							
5. Consequence distortions	2.05	1.05	0.32**	0.37**	0.69**	0.43**	–						
6. Advantageous comparison	1.93	1.06	0.52**	0.51**	0.39**	0.41**	0.31**	–					
7. Diffusion of resp.	2.44	1.06	0.47**	0.46**	0.46**	0.49**	0.34**	0.33**	–				
8. Victim dehum.	2.00	0.87	0.36**	0.39**	0.47**	0.67**	0.39**	0.39**	0.37**	–			
9. Moral dis. total score	2.19	0.70	0.73**	0.74**	0.72**	0.76**	0.58**	0.63**	0.74**	0.65**	–		
10. Bullying	1.88	0.71	0.46**	0.42**	0.30**	0.30**	0.27**	0.33**	0.36**	0.27**	0.48**	–	
11. Gender	–	–	-0.20**	-0.19**	-0.15**	-0.09**	-0.18**	-0.16**	-0.14**	-0.14**	-0.21**	-0.28**	–


*^∗∗^*p* < 0.001*.

**Table 2:** Means, standard deviation, and correlations, separately for boys and girls.

	Boys		Girls											
	Mean	*SD*	Mean	*SD*	1	2	3	4	5	6	7	8	9	10
1. Moral justification	2.60	1.05	2.16	0.98	–	0.56**	0.41**	0.45**	0.29**	0.50**	0.44**	0.34**	0.73**	0.44**
2. Euphemistic labeling	2.21	0.10	1.84	0.86	0.62**	–	0.43**	0.48**	0.33**	0.47**	0.42**	0.39**	0.74**	0.40**
3. Blaming the victim	2.33	0.98	2.05	0.90	0.43**	0.46**	–	0.75**	0.68**	0.38**	0.41**	0.44**	0.70**	0.28**
4. Displacement resp.	2.40	0.92	2.24	0.88	0.44**	0.47**	0.77**	–	0.40**	0.44**	0.47**	0.65**	0.77**	0.30**
5. Consequence distortions	2.25	1.09	1.87	0.96	0.30**	0.37**	0.69**	0.44**	–	0.29**	0.31**	0.35**	0.52**	0.25**
6. Advantageous comparison	2.10	1.11	1.65	0.78	0.50**	0.52**	0.37**	0.37**	0.29**	–	0.30**	0.41**	0.65**	0.29**
7. Diffusion of resp.	2.60	1.10	2.29	1	0.48**	0.47**	0.48**	0.50**	0.33**	0.32**	–	0.33**	0.70**	0.37**
8. Victim dehum.	2.13	0.91	1.88	0.81	0.34**	0.37**	0.47**	0.68**	0.39**	0.35**	0.37**	–	0.65**	0.21**
9. Moral dis. total score	2.30	0.72	1.99	0.64	0.73**	0.76**	0.73**	0.76**	0.54**	0.64**	0.73**	0.65**	–	0.46**
10. Bullying	2.08	0.75	1.69	0.60	0.42**	0.38**	0.28**	0.29**	0.23**	0.32**	0.31**	0.27**	0.45**	–


*^∗∗^*p* < 0.001. Correlations for boys are reported below the diagonal, correlations for girls are reported above the diagonal*.

### Relations Between Moral Disengagement Mechanisms and Bullying: Structure Equation Modeling

**FIGURE 1:**
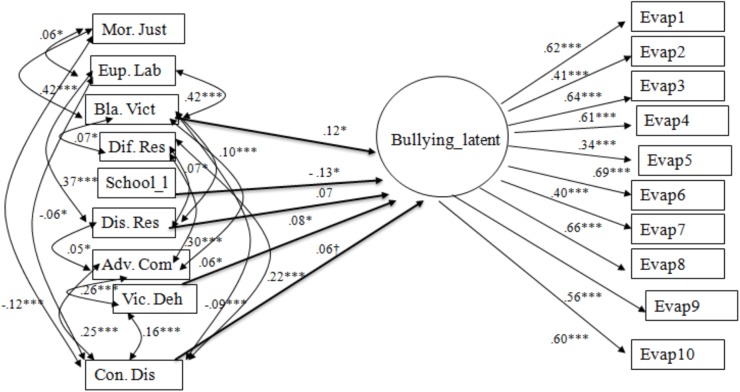
Overall Group Model of Associations between Bullying Behavior and Moral Disengagement Mechanisms. Mor.Just, Moral justification; Eup. Lab, Euphemistic labeling; Adv. Com, Advantageous comparison; Dis. Res, Displacement of responsibility; Dif. Res, Diffusion of responsibility; Con. Dis, Consequence distortions; Bla. Viet, Blaming the victim; Vic. Deh, Victim Dehumanization; SchoolJ, school level. Non-significant paths are not shown in the figure. ^†^Significance is marginal. ^∗^*p* < 0.05, ^∗∗^*p* < 0.01, ^∗∗∗^*p* < 0.001.

When tested in the overall group, the model (Figure 1) fitted the data adequately [χ^2^(116) = 250.48, *p* < 0.001, *CFI* = 0.94, *RMSEA* = 0.031] and bullying behavior was positively associated with the mechanisms of Blaming the victim (0.12, *p* < 0.05), Victim dehumanization (0.08, *p* < 0.05), Displacement of responsibility (0.07, *p* = 0.05), and, marginally, with Consequence distortions (0.06, *p* = 0.055). Bullying was also negatively associated with age (assessed by the school level; -0.13, *p* < 0.05).

**FIGURE 2:**
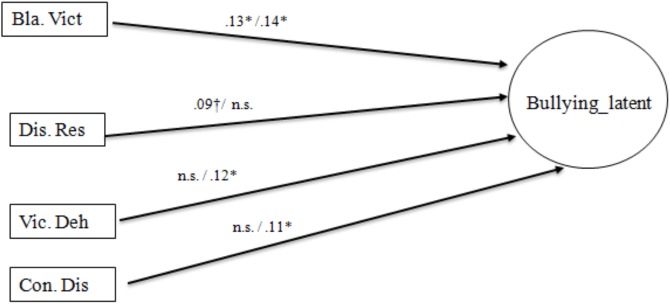
Multigroup model of Associations between Bullying Behavior and Moral Disengagement Mechanisms. Dis. Res, Displacement of responsibility; Con. Dis, Consequence distortions; Bla. Viet, Blaming the victim; Vic. Deh, Victim Dehumanization; Non-significant paths are not shown in the figure. In each path, male group values are shown on the left, female group values on the right. ^†^Significance is marginal. ^∗^*p* < 0.05, ^∗∗^*p* < 0.01, ^∗∗∗^*p* < 0.001.

The model fitted the data adequately also among both the gender groups: boys, χ^2^(116) = 174.25, *p* < 0.001, *CFI* = 0.95, *RMSEA* = 0.029; girls, χ^2^(116) = 193.38, *p* < 0.001, *CFI* = 0.92, *RMSEA* = 0.033. The fit of the multiple group model for the gender groups, where the invariance of the measure for the bullying factor was controlled for, was also good: χ^2^(250) = 454.54, *p* < 0.001, *CFI* = 0.94, *RMSEA* = 0.031. The paths emerging in the two groups as estimated in the multiple group model are reported in Figure 2. Among boys, bullying was linked to the Blaming the victim mechanism (0.13, *p* < 0.05), and, marginally (*p* = 0.07), to Displacement of responsibility (0.09). Among girls, variation in bullying was significantly predicted by the mechanisms of Blaming the victim (0.14, *p* < 0.05), Victim dehumanization (0.12, *p* < 0.05), Consequence distortions (0.11, *p* < 0.05).

### The Spontaneous Use of Moral Disengagement: Qualitative Analysis

The results of the qualitative analysis provided confirmation to the results of the quantitative analysis. Focusing in the frequency of behaviors, 8 adolescents of the 55 interviewed participants reported to have act bullying behaviors (bullies), while 42 stated that they witnessed bullying situations (bystanders). Moreover, seven adolescents declared no involvement at all in bullying situations. In the narratives, mentions of moral disengagement mechanisms emerged for both boys and girls (Table 3). The way mechanisms were reported was very similar across genders, as was the type of involvement (as bully or bystander) in bullying situations.

**Table 3:** Definition and examples of the moral disengagement mechanisms emerged spontaneously in the interviews.

Moral disengagement mechanisms	Explanation (Bandura, 2002)/Example	Number of occurring^∗^ in interviews by gender and type of involvement in bullying			
		Girls (*N* = 25)		Boys (*N* = 30)	
		Bullies (*N* = 2)	Bystanders (*N* = 8)	Bullies (*N* = 6)	Bystanders (*N* = 16)
Blaming the victim	Believing that the victim deserves his or her suffering. Examples: “She is a girl who does not talk to anyone. Therefore, everyone was importuning her. Me too.” (Girl 38, 17 years). “I excluded a boy because he was irritating.” (Boy 18, 13 years).	3	10	4	11
Consequence distortions	Minimizing, ignoring, or misconstruing the negative or harmful effects of actions. Examples: “Another time I said to the girls [bullies] to stop what they were doing because they were going to be harmed.” (Girl 21, 12 years). “It is normal because we never got into a real fight” (Boy 10, 15 years).	1	1	5	1


The use of moral disengagement mechanisms was not highlighted in all the interviews, but emerged spontaneously in several of them. Overall, common justifications for bullying, which were provided by both bullies and bystanders’ in their interviews, were that victims are “different” in comparison to the other peers, and that the victims tended to behave in ways that were considered negatively by peers (e.g., “she/he does not talk to anyone, does not interact with peers”). These responses reflect the moral disengagement mechanisms of dehumanization of the victims and blaming the victim. Many of the adolescents who were interviewed also showed tendencies to underestimate the negative outcomes for the victim (consequence distortions), to diluite the personal responsibility of bullying within the peer group (diffusion of responsibility), and to consider bullying as a game (euphemistic labeling) (Table 3).

Boys, compared to girls, used a greater number of moral disengagement mechanisms. Girls mainly resort to the mechanism of blaming the victim, in order to justify their action. Boys, in addition to blaming the victim, described bullying as a game, highlighting euphemistic labeling processes. Furthermore, boys minimized the negative effects of actions, and justified bullying by appealing to moral principles. In some cases, participants, evaluated bullying as “trivial.” Boys also reported feelings of apathy or indifference when witnessing aggression. For instance, common boys’ responses were: “I did not feel anything after the aggressions” or “it is normal.”

In addition, when bystanders acknowledged the seriousness of the problem, they also reported how they didn’t try to help or support the victims. Some participants mentioned that sometimes they tend not to engage in or to withdrew from bullying situations in order to avoid becoming victims themselves. These responses suggest a complex interplay between bullying, emotions (such as fear, apathy, and indifference), and morality in the Brazilian context.

In general, besides moral disengagement mechanisms, a multifaceted picture of the associations between moral dimensions and bullying emerged from the participants’ narratives. Issues of hedonism, of pursuing personal goals and prioritizing personal interests as motives for bullying could be found in the narratives (e.g., “I think that I just didn’t like him when I was a child. He reminded me of the myself”). In addition, in bystanders’ interviews, issues of moral engagement were identified, such as the wish to express solidarity for the victims, tolerance of diversity, reference to human rights, otherness, non-violence culture and antibullying attitudes (e.g., “I told them to stop. I said: “Remember *Do unto others as you would have them do unto you*. We have to treat people in the same way we want to be treated” ”).

## Discussion

The main aim of this study was to explore the associations between moral disengagement processes and bullying among Brazilian adolescents (aged 10–19 years).

As a first step, we investigated the association between bullying and moral disengagement processes as derived from quantitative data. We hypothesized that among Brazilian adolescents the use of moral disengagement to self-justify bullying would have been associated with higher levels of bullying, confirming data reported in international literature (Caravita et al., 2012).

Our results confirm that moral disengagement, as a stable trait, is associated with bullying behavior also among Brazilian youths. The exam of both qualitative and quantitative data confirmed that Brazilian adolescents use moral disengagement mechanisms to self-justify bullying. These findings are in line with the data from international studies across different countries, such as Canada (Hymel et al., 2005), Italy and Spain (Menesini et al., 2003), Sweden (Thornberg and Jungert, 2014), and Denmark (Obermann, 2011). In all these contexts, adolescents who can be identified as bullies have been found to resort to higher levels of self-justification processes.

As a second step, we tested the hypothesis that spontaneous use of moral disengagement mechanisms to explain bullying and the passive bystander behavior could be identified using qualitative data to analyze adolescents’ spontaneous narrative of bullying behaviors. Our qualitative data highlighted how that the use of self-justification processes could be observed in some bystanders’ statements. One of the most prominent result, clearer when analyzing boys’ interviews, was the tendency to describe bullying as “trivial” or “normal.” Generally speaking, emotions of apathy, and indifference for witnessing or acting bullying emerged from the narratives. Menesini et al. (2003) suggest that these emotions are typically related to moral disengagement. Even if we could not explore the relation between the bystander passive behavior and the moral disengagement processes using a quantitative approach, these cognitive and emotional reactions reported in the interviews, indicate that Brazilian adolescents who are involved in bullying as outsiders can resort to self-justifications to avoid moral feelings, such as guilt, regret or shame. Altogether, our data on the pervasive use of self-justifying processes among bullies and bystanders support the conceptualization of moral disengagement as subtly diffuse in the society (Bandura, 2002), and as a cognitive process that is shared within the social contexts where adolescents live (Caravita et al., 2014). The current study provides evidence on how this conclusion is valid for the Brazilian society too. The consistency of our findings with the ones from the international literature suggests a possible universality of these associations, even if cultural specificities may emerge in the use of specific moral disengagement mechanisms.

Our third goal was to examine whether distinct associations of the eight moral disengagement mechanisms with involvement in bullying as a bully (both quantitative and qualitative data) and as a passive bystander (qualitative data) exist. By examining the mechanisms of moral disengagement separately, we found that not all the mechanisms were associated with bullying or were reported in the interviews in relation to the bystander behaviors. Both the qualitative and the quantitative results showed that the use of blaming the victim to justify bullying situations was predominant. The bullying behavior was also associated with the mechanisms of victim dehumanization, displacement of responsibility, and consequence distortions. In the interviews, however, the euphemistic labeling was also used, as well as the mechanisms of moral justification and diffusion of responsibility. When examined in comparison to the findings from other cultural contexts, our outcomes may highlight some cultural specificity of the Brazilian culture. For instance, in a study involving 339 United Kingdom students (7–9 years) Pornari and Wood (2010) found that higher levels of moral disengagement were positively associated to traditional and cyber-aggression, but only moral justification, euphemistic labeling, and displacement of responsibility predicted significantly and positively the variance of traditional aggression. These mechanisms only partially overlap with the mechanisms that emerged as connected to the bullying behavior among Brazilian adolescents from the quantitative data. In the Brazilian context, the stable tendency to use self-justification processes of moral justification and euphemistic labeling was not significantly connected with bullying behaviors, while higher levels of dehumanizing the victim and minimizing the consequences of the behavior were. As mentioned in the Introduction, the Brazilian society is characterized by a large diversity for ethnicity and cultural background, and by high levels of internal migration. The large differences existing in the population composition are also connected to tensions among the population components to a higher extent than in other cultural contexts. This factor may favor the diffusion and the use, among Brazilian adolescents, of more explicit types of self-justification processes, where the reasons for the aggression are found in differences and in the status of the victim, perceived as less worthy (victim dehumanization), or where the seriousness of the aggression is denied. Hence, some cultural specificity may exist, and this might be even connected with differences in the evaluation of specific forms of behavior as immoral conduct across the different cultural contexts (Menesini et al., 2003; Obermann, 2011).

It should be also considered, however, that the use of moral justification and euphemistic labeling to explain bullying emerged from the adolescents’ interviews, even if these mechanisms were not associated to the bullying behavior in the quantitative data. The interviews may have allowed highlighting the use of moral disengagement mechanisms that are less strongly associated to the behavior, when it is assessed as stable dispositions. That is to say that, even these adolescents who do not have a stable tendency to resort to these mechanisms, can use these forms of self-justifications when they are asked to talk about their bullying experiences and, when the request indirectly allows to better elaborate their thinking about what happened, as it is the case with an interview.

The displacement of responsibility mechanism also appeared to be associated with the bullying behavior, and expressions of self-justification processes related to the diffusion of responsibility were mentioned in the interviews. Both displacement and diffusion of responsibility are processes that are directly connected with the presence of the group. This is consistent with the consideration that bullying is a group phenomenon and cannot be conceived only as an individual issue (Caravita et al., 2011b; Hymel and Bonanno, 2014). Hence, bullying cannot be understood without considering also the peer group (e.g., Salmivalli et al., 1996), and for this reason it is not surprising that when they are (self-) justifying their behaviors, bullies use also mechanisms where the responsibility of the action can be attributed to another peer or diluted within the peer-group (Caprara et al., 1995). Furthermore, there is evidence to support the fact that the use of moral disengagement is largely influenced and connected to the group dynamics. Moral disengagement justifications can be socialized among peers, especially in adolescence (Caravita et al., 2014). Furthermore, within the bullying process the group members can share a negative perception of the victim (Gini, 2008) and they can share evaluations of the aggressive behaviors as less or more admissible, in light of the peer-group informal norms (Chang, 2004).

Lastly, we hypothesized that gender may moderate the associations between mechanisms of moral disengagement, bullying and bystander behaviors, and that boys may be more prone to self-justifications than girls. Findings from this study showed that some differences in the use of moral disengagement mechanisms across genders can be found. Both boys and girls used (self-) justifications practices consisting in blaming the victims. The mechanism of blaming the victim was also the type of justification for bullying that was the most mentioned in the interviews by both the gender-groups. Both boys and girls appeared not to perceive the suffering of the victim, and tended to attribute the blame of the detrimental behaviors to the victims themselves (victim blame mechanism). However, in the interviews, boys also reported statements where bullying was justified on the basis of moral values (moral justification), or where bullying was described as a game (euphemistic labeling mechanisms), and showed a general tendency to minimize the consequences of bullying. Moreover, only among boys the displacement of responsibility was related to the bullying behavior, even if marginally. In contrast, only among girls the tendencies to dehumanize the victim and to minimize the consequences of the bullying behavior were related to bullying.

These gender differences suggest that boys resort to the support of the group, and share the responsibility for the detrimental actions with the other peers more than girls do. This finding may also highlight some differences in the relevance that boys and girls attribute to the group status and dynamics. Teenagers boys might be more concerned about peer-group dynamics and their status within the group (Pouwels et al., 2015). They might also live the group more intensely than girls do, and hence use bullying to acquire and keep a prominent status, and to establish social dominance hierarchies within the group (Turner, 2009). Following this line of reasoning, boys will then resort to the group itself to self-justify their bullying behavior. In contrast girls, who can rely on themselves more than boys and give to the group a less relevance than boys, may resort more frequently to self-justification mechanisms by minimizing the action consequences. Girls, more than shifting the responsibility on the group, may then underestimate the severity of the victimization, and end up dehumanizing the victim. Further studies are needed to examine specific gender differences related to the use of moral disengagement processes and to the involvement in bullying situations, in order to confirm our results.

Our findings suggest that the use of mixed method can allow a better identification of the moral disengagement mechanisms that are associated with the bullying behavior and the passive bystander behavior. Indeed, as told above, only in the interviews it was possible to highlight some of the self-justification processes that are used to explain the personal behavior in bullying situations. In the results derived from the quantitative data, the moral justification and the diffusion of responsibility processes were not related to bullying; yet, when the adolescents’ narratives of their personal experiences in bullying were examined these mechanisms clearly emerged. Hence, qualitative data allowed deriving some information about bullying and the use of self-justifications, that quantitative data alone would not have highlighted. Accordingly, other studies indicate that combining quantitative and qualitative methods is a good strategy to better understand the dynamics of bullying as a social phenomenon and its relations with morality issues (Thornberg and Jungert, 2014; Thornberg et al., 2014).

Qualitative data also allow highlighting more clearly how the students actually recognize how bullying behaviors are negative, and how they, as a direct consequence, resort to specific mechanisms of cognitive restructuring of the meaning of their behaviors to self-justify their actions. As a final note, the qualitative data seem to allow examining directly a “live” use of moral disengagement act by teenagers to justify bullying. This analysis appears to add a new depth to the information derived from quantitative data.

Some limitations of this study need to be considered. First, our quantitative data was cross-sectional, thus we could not identify causal associations between the variables. Second, the measure of bullying was self-report and did not allow assessing the bystander roles of participation in bullying. Although self-report measures are often used to assess behaviors in bullying situations, this type of measure can be affected by social desirability effects. With regard to bystanders, they play a relevant role in the bullying dynamics and also when planning target anti-bullying interventions (Salmivalli, 2014; Thornberg et al., 2012b). Thus, for future research investigating bullying in Brazil, planning a methodological approach that takes into consideration multiple source of information and especially also assesses bystanders’ behavior has to be recommended in order to provide a broader view on this issue. Future studies on this topic may introduce longitudinal designs to clarify the causal associations among the variables examined in the quantitative data. Regarding results derived from the qualitative data, they are relevant and innovative, but they may reflect only a process specific to the Brazilian sample targeted by this study. For these reasons, more research is needed to investigate the themes that emerged from the presented data. Likewise, another limitation refers to the risk that the students’ responses during the interviews could have been influenced by biases related to previous exposure to the quantitative measures.

Despite these limitations, this study extends the knowledge of school bullying in Brazil and its relations with moral disengagement construct, and with moral dimensions in general. To our knowledge, this is the first study to examine processes of self-justification associated with bullying among Brazilian adolescents. As further strength, we distinguished between the different moral disengagement processes and we investigated their associations with bullying and the bystander behaviors separately. The large sample and the sampling procedure also strengthened the representativeness of our data.

In addition, the mixed method approach allowed us to derive more detailed information on the morality-related processes that are associated to a higher risk for bullying in schools, and to explore more in depth the mechanisms of moral disengagement within bullying situations. The use of self-justification processes related to bullying emerged spontaneously in adolescents’ narratives. From a methodological perspective, findings from this study highlight the value of adopting qualitative methods to investigate intra-psychological mechanisms, such as moral disengagement. With regards to interventions to address bullying, this outcome, in line with the findings from the quantitative analysis, provides a stronger evidence of the relevance of these psychological processes as possible risk factors for bullying. However, not all the self-justifications processes were linked to bullying and emerged in the narratives, and some gender-differences emerged as well. These differences should be taken into account in order to develop actions to tackle the specific mechanisms that may favor bullying or a passive bystander behavior. As suggested by the findings on moderations by gender, different actions should be also be developed to address types of self-justifications according to gender, since boys and girls act differently when self-justifying bullying or their avoiding to help the victims when they act as bystanders. Lastly, studies on bullying in Brazil are in an early phase, and there is a specific need to individuate and to develop specific interventions that are likely to be successful in this cultural context. These interventions should try to directly address the psychological processes that increase the risk of acting or supporting bullying behaviors. Findings from this study support the view that also in Brazil, not only bullying is a complex phenomenon, but it is also linked to some moral distortions. We need further studies to confirm this result, but it is likely that anti-bullying intervention addressing these morality distortions, and fostering the moral engagement of adolescents, can result effective in preventing and, possibly reducing bullying in Brazilian schools too. These interventions need to consider the cultural specificities of bullying in Brazil, and of the related morality issues. Increasing, through these interventions, the levels of moral engagement, measured by tolerance for diversity, solidarity and ethics in relations, may contribute not only to fight aggression among peers but also to build a society based on non-violence.

## Author Contributions

WO contributed substantially to the conception of the study, the development of the measures, the data collection, the data analysis, and the interpretation of the results, and to writing the article. SC contributed substantially to the conception of the study with the focus on moral disengagement, the analysis of the quantitative data, the interpretation of the results, and contributed to drafting the article. BC contributed substantially to writing the article, in particular to drafting the introduction. ED contributed substantially to the interpretation of the results and drafting the article. JS contributed substantially to the data collection and the analysis of the qualitative data. MS contributed substantially to the conception of the study, the data collection and analysis, and leads the research of the Brazilian group on this topic.

## Conflict of Interest Statement

The authors declare that the research was conducted in the absence of any commercial or financial relationships that could be construed as a potential conflict of interest.
